# Correlates of occupational, leisure and total sitting time in working adults: results from the Singapore multi-ethnic cohort

**DOI:** 10.1186/s12966-017-0626-4

**Published:** 2017-12-13

**Authors:** Léonie Uijtdewilligen, Jason Dean-Chen Yin, Hidde P. van der Ploeg, Falk Müller-Riemenschneider

**Affiliations:** 1 Saw Swee Hock School of Public Health, National University of Singapore, Singapore, Singapore; 2 Department of Public & Occupational Health, Amsterdam Public Health Research Institute, VU University Medical Center, Amsterdam, the Netherlands; 3 Sydney School of Public Health, University of Sydney, Sydney, NSW Australia; 4 Institute for Social Medicine, Epidemiology and Health Economics, Charite University Medical Centre Berlin, Berlin, Germany; 5 Saw Swee Hock School of Public Health and Department of Medicine, Yong Loo Lin School of Medicine, National University of Singapore, Tahir Foundation Building, 12 Science Drive 2, #10-01, Singapore, 117549 Singapore

**Keywords:** Sitting, Adults, Asia, Multi-ethnic, Prevalence, Correlates

## Abstract

**Background:**

Evidence on the health risks of sitting is accumulating. However, research identifying factors influencing sitting time in adults is limited, especially in Asian populations. This study aimed to identify socio-demographic and lifestyle correlates of occupational, leisure and total sitting time in a sample of Singapore working adults.

**Methods:**

Data were collected between 2004 and 2010 from participants of the Singapore Multi Ethnic Cohort (MEC). Medical exclusion criteria for cohort participation were cancer, heart disease, stroke, renal failure and serious mental illness. Participants who were not working over the past 12 months and without data on sitting time were excluded from the analyses. Multivariable regression analyses were used to examine cross-sectional associations of self-reported age, gender, ethnicity, marital status, education, smoking, caloric intake and moderate-to-vigorous leisure time physical activity (LTPA) with self-reported occupational, leisure and total sitting time. Correlates were also studied separately for Chinese, Malays and Indians.

**Results:**

The final sample comprised 9384 participants (54.8% male): 50.5% were Chinese, 24.0% Malay, and 25.5% Indian. For the total sample, mean occupational sitting time was 2.71 h/day, mean leisure sitting time was 2.77 h/day and mean total sitting time was 5.48 h/day. Sitting time in all domains was highest among Chinese. Age, gender, education, and caloric intake were associated with higher occupational sitting time, while ethnicity, marital status and smoking were associated with lower occupational sitting time. Marital status, smoking, caloric intake and LTPA were associated with higher leisure sitting time, while age, gender and ethnicity were associated with lower leisure sitting time. Gender, marital status, education, caloric intake and LTPA were associated with higher total sitting time, while ethnicity was associated with lower total sitting time. Stratified analyses revealed different associations within sitting domains for Indians compared to Chinese and Malays.

**Conclusion:**

Our findings highlight the need to focus on separate domains of sitting (occupational, leisure or total) when identifying which factors determine this behavior, and that the content of intervention programs should be tailored to domain-specific sitting rather than to sitting in general. Finally, our study showed ethnic differences and therefore we recommend to culturally target interventions.

## Background

The science of sitting and sedentariness, which comprises research on behaviors such as TV viewing, sitting at work and sitting for transport, is considered an emerging public health field. Evidence on the health risks of sitting is accumulating, with studies among large cohorts of adults showing detrimental effects of sitting on all-cause mortality [1–3], cardio metabolic risk profiles [1, 4–6] and mental health [7]. Too much sitting can thus be hazardous to population health, even though its health implications seem to be attenuated by physical activity participation [8].

Similar to trends in the US and the UK, sitting time of Asian adults, such as Chinese and Indians, is predicted to rise over the next decade [9]. Moreover, current research among adults from other countries in Asia found that they engage in high levels of sitting too. For example, results from a Singapore population-based study showed that 37% of the 2319 participants included in the study, sat for at least 8 h a day [10]. These figures correspond with another study done in Japan, in which adults’ self-reported average sitting time was 8.4 h a day, and accelerometer data collected among the same participants showed an average sitting time of 8.8 h a day [5].

Recent research among both Western and Asian working adults found that occupational sitting time contributed greatly toward daily sitting time [11, 12]. In modern, technologically advanced and automated societies, it is no surprise that adults spend a large proportion of their working time sitting at their desks or in meetings. Subjective and objective assessment of sitting in office workers further showed that participants accumulated higher levels of sitting time on working days compared to non-working days [13–15], and that they had fewer breaks from sitting during working hours compared to non-working hours [16]. It is still largely unclear whether occupational sitting time has a more profound effect on health than e.g., leisure time sitting [17, 18], which is a more frequently examined domain of sitting. Nevertheless, the workplace could be a key setting in which to reduce adults’ sitting time and occupational sitting may therefore be of specific interest to researchers around the globe.

To support the development of effective intervention programs to reduce sitting time, O’Donoghue and colleagues [19] conducted a comprehensive systematic literature review summarizing the evidence on individual, social, environmental and policy-related correlates and determinants of sitting in adult populations. They reported that individual level factors such as older age, low physical activity levels, high body mass index (BMI) and female gender have often been examined and all seem to be associated with having higher sitting time. Yet, of the 74 studies that were included in this review, the vast majority was based on data from the US, Europe or Australia. Only four studies were conducted in Asia: three in Japan and one in Hong Kong. Research on sitting has predominantly been done in Western cohorts, and the emphasis has largely been on assessing sitting prevalence and/or its health effects. To further progress this field, research needs to focus on more diverse, non-Western populations [20], and the identification of factors influencing sitting behaviors.

In this context, the research aims of the current study are:

- to describe the socio-demographic and lifestyle correlates of different domains of sitting, namely occupational, leisure and total sitting in a large cohort from Singapore;
- to investigate potential differences in correlates of sitting time among Chinese, Malay and Indian sub-groups; and
- to explore, in light of the suggested non-linear relationship of sitting and health risks, whether correlates of sitting time are consistent for continuous versus dichotomous (low/high) outcomes.

## Methods

The Multi-Ethnic Cohort (MEC) was formed by amalgamating two existing population-based cohorts recruited between 2004 and 2007: the Singapore Prospective Study Program (SP2) and the Singapore Cardiovascular Cohort Study (SCCS2) [21, 22], with additional recruitment of participants from 2007 to 2010. The SP2 and SCCS2 recruited 8340 participants from four previous cross-sectional studies: Thyroid and Heart Study 1982–1984, National Health Survey 1992, National University of Singapore Heart Study 1993–1995 and National Health Survey 1998. All four studies involved a random sample of Singapore adult residents, with oversampling of ethnic minorities, i.e. Malays and Indians. Detailed information on the MEC can be found on http://blog.nus.edu.sg/sphs/.

In addition to the participants from SP2 and SCCS2, a further 6125 Singapore residents were recruited into the MEC study through public outreach and referrals from existing cohort members. Invitation to participate was opened to any Singapore citizens or long-term residents aged 21 to 75 years old. Recruitment drives were carried out at community events, mosques and temples to enrich the proportion of Malay and Indian participants. Due to the complex nature of the different recruitment strategies, tracking of response and eligibility were not feasible. People who have or had heart disease, stroke, cancer and renal failure were excluded from participation in the MEC. Furthermore, for this study, participants who were not working over the past 12 months (*n* = 5088) or had missing data on working status (*n* = 8) were excluded from the analyses. Those without data on sitting time (*n* = 4) were excluded from the analyses too.

### Data collection and outcome measures

All MEC participants were visited and interviewed at home by trained interviewers. The approximately one-hour long interview included information on socio-demographic details, medication use, medical history, family history of disease, environmental tobacco exposure, use of tobacco and alcohol, diet, physical activity, sitting time and health-related quality of life. Participants were also invited to attend a physical examination, which included blood and urine collection.

#### Dependent variables: sitting time

Questions on sitting time were modified from the Modifiable Activity Questionnaire [23, 24] and the Minnesota Leisure Time Activity Questionnaire [25]. Participants reported the average hours per day they spent sitting down while at work over the past three months, and during leisure time on week days and weekend days. Occupational sitting time was derived for each occupation reported by the participants, acknowledging that one person may hold two or more jobs. The reported number of hours was multiplied by the number of days per week worked – for that specific job – and then divided by 7. Occupational sitting time was calculated for both part-time and full-time workers. Leisure sitting time was calculated by collating the time spent sitting during free time on week days and weekend days. Values were weighted (5× for week day sitting and 2× for weekend day sitting, divided by 7) to get an accurate estimate for leisure sitting time per day. Total sitting time was calculated by summing the values from occupational and leisure sitting. For the current study, all domains of sitting were expressed in hours per day.

#### Independent variables: socio-demographics and lifestyle

Socio-demographic variables were self-reported by questionnaire and included age (21–30, 31–40, 41–50, 51–60, 61+ years), gender (males, females), ethnicity (Chinese, Malay, Indian), marital status (married, never married, separated/divorced/widowed), and level of education (no formal or primary education, secondary education, A’level, university and above). Lifestyle variables were also self-reported by questionnaire and included smoking, caloric intake derived from diet and leisure time physical activity (LTPA).

Smoking status was sorted into three groups: non-smokers, former smokers, and current smokers. Non-smokers were categorized as those who had never smoked in their life, or who had previously smoked, but not for 30 days or more continuously. Former smokers were those who had smoked for 30 days or more continuously in the past, but had since quit. Current smokers were those that reported that they were currently smoking.

Physical activity was assessed with the SP2 Physical Activity questionnaire (SP2PAQ), which was adapted from several established questionnaires validated in other populations [26]. The SP2PAQ has a recall period of the previous 3 months and encompasses transportation, occupation, leisure time and household activities. For the current study, we only included moderate-to-vigorous LTPA, which has been used widely as a general indicator of physical activity. To categorize participants, those with no LTPA were clustered in the ‘0’ group, while remaining participants who did engage in LTPA were split into two groups based on the median value as calculated in MET-h/week (see Table 1).

To assess diet, the MEC questionnaire utilized a food frequency questionnaire (FFQ) that was validated in the Singaporean context [27]. The FFQ asked about past month consumption frequency of a large variety of food groups, including rice, noodles, vegetables and bean curd, meat, seafood, desserts and local snacks etc. From the raw data, total caloric intake (Kcal/day) was computed. To determine the cut-offs for consumption amounts, we used quartiles and split the data into four groups for each gender separately, accounting for sex differences in caloric consumption. Values of quartiles for males were (in Kcal/day): 550.56 to 1791.14 (1st quartile), 1791.14 to 2277.51 (2nd quartile), 2277.51 to 2910.28 (3rd quartile), 2910.28 to 19,465.12 (4th quartile). Values of quartiles for females were: 96.43 to 1500.88 (1st quartile), 1500.88 to 1919.83 (2nd quartile), 1919.83 to 2460.16 (3rd quartile), 2460.16 to 16,449.34 (4th quartile).

### Statistical analysis

Descriptive statistics (mean and SD, or proportions) were derived for all dependent and independent variables (Table 1). Missing data were only present in the marital status (*n* = 4), education (*n* = 2) and smoking (*n* = 1) variables, and were kept as missings in the analyses. All sitting time distributions were checked and diagnosis plots revealed a slight right-skew. Due to the large sample size and variation, along with homoscedasticity, non-normality of outcome variables was considered negligible and sitting time was not transformed in the analyses. We tested for effect modification by ethnicity (data not shown), and based on these findings, multivariable regression models were used to examine associations between socio-demographics and lifestyle and all domains of sitting time, for the entire sample and subsequently stratified for Chinese, Malay and Indian participants. Additional multivariable regression models were used to examine the association between socio-demographic and lifestyle factors and dichotomized sitting time, i.e., for the ‘low’ sitting versus the ‘high’ sitting group. Cut-offs were taken from Chau et al. [2]: <8 h/day versus ≥8 h/day for total sitting time and <4 h/day versus ≥4 h/day for occupational and leisure sitting time since they contributed roughly equal parts to total sitting time. All multivariable models were corrected for the independent variables listed above, as well as total working time in hours per day. For example, the association of age with occupational sitting time was adjusted for gender, ethnicity, marital status, education, smoking, diet, physical activity and total working time. To assess whether there were ethnic differences between ‘low’ and ‘high’ sitting groups (using the cut-offs above), chi-squared tests were used. Participants of non-Chinese, non-Malay and non-Indian ethnicity (*n* = 31) were retained in the total sample analyses, but results were not presented in the main analyses tables (Tables 2, 3, 4 and 5) due to their small numbers. To test the robustness of our results, all multivariable models were repeated for participants who were considered to work full-time, i.e., ≥38 h/wk. (*n* = 8034). Results from these analyses were compared with the results of the original sample (*n* = 9384), which also included part-time workers.

To check for selection bias, we compared the full MEC dataset (*n* = 14,476) with our final sample on key socio-demographic variables using chi-squared tests. All analyses were conducted with R statistical software version 3.4.1, and findings were considered statistically significant if *p* < 0.05.

## Results

Table 1 provides descriptive information on the final sample of 9384 participants (54.8% male, 50.5% Chinese, 24.0% Malay, and 25.5% Indian), including average sitting time for occupational, leisure and total sitting according to categorized socio-demographic and lifestyle variables. Selected participants were younger, more often male, Chinese and married, and had a higher education (all *p* < 0.001) than excluded participants (data not shown).

**Table 1:** Participant characteristics and descriptive information on sitting time

	n	%	Occupational sitting in h/day	Leisure sitting in h/day	Total sitting in h/day
			(Mean ± SD)	(Mean ± SD)	(Mean ± SD)
Age					
21–30	1437	15.3	2.70 ± 2.19	3.13 ± 1.59	5.83 ± 2.62
31–40	2308	24.6	2.94 ± 2.19	2.83 ± 1.43	5.77 ± 2.57
41–50	3247	34.6	2.76 ± 2.34	2.64 ± 1.39	5.40 ± 2.69
51–60	1913	20.4	2.54 ± 2.34	2.67 ± 1.51	5.21 ± 2.74
61+	479	5.1	2.10 ± 2.28	2.57 ± 1.35	4.67 ± 2.57
Gender					
Male	5142	54.8	2.73 ± 2.33	2.79 ± 1.47	5.53 ± 2.70
Female	4242	45.2	2.69 ± 2.23	2.73 ± 1.46	5.42 ± 2.63
Ethnicity					
Chinese	4722	50.5	3.03 ± 2.33	2.79 ± 1.47	5.82 ± 2.70
Malay	2246	24.0	2.36 ± 2.16	2.85 ± 1.46	5.21 ± 2.55
Indian	2385	25.5	2.42 ± 2.23	2.63 ± 1.46	5.05 ± 2.64
Other	31	0.3	2.96 ± 2.36	2.81 ± 1.29	5.77 ± 2.72
Marital Status					
Married	6962	74.2	2.73 ± 2.31	2.68 ± 1.43	5.41 ± 2.67
Never Married	1889	20.1	2.86 ± 2.22	3.05 ± 1.53	5.91 ± 2.63
Separated/Divorced/Widowed	529	5.6	2.00 ± 2.14	2.81 ± 1.60	4.81 ± 2.64
Highest education					
No Formal/PSLE	1903	20.3	1.70 ± 2.16	2.71 ± 1.55	4.41 ± 2.59
Secondary/O/N/ITE/NTC	4094	43.6	2.64 ± 2.31	2.75 ± 1.40	5.39 ± 2.64
A’level/Polytech/Diploma	1891	20.2	3.12 ± 2.04	2.84 ± 1.49	5.95 ± 2.50
University and above	1494	15.9	3.71 ± 2.11	2.77 ± 1.48	6.49 ± 2.55
Smoking					
No	6762	72.1	2.82 ± 2.26	2.71 ± 1.41	5.53 ± 2.63
Former	756	8.1	2.72 ± 2.34	2.76 ± 1.51	5.48 ± 2.76
Yes	1865	19.9	2.33 ± 2.33	2.97 ± 1.61	5.30 ± 2.77
Total caloric intake (Kcal/day)					
1st quartile	2347	25.0	2.54 ± 2.15	2.72 ± 1.22	5.25 ± 2.40
2nd quartile	2345	25.0	2.68 ± 2.32	2.70 ± 1.38	5.38 ± 2.59
3rd quartile	2345	25.0	2.86 ± 2.30	2.77 ± 1.53	5.64 ± 2.78
4th quartile	2347	25.0	2.78 ± 2.37	2.87 ± 1.68	5.65 ± 2.86
Moderate-to-vigorous LTPA (MET-h/week)					
0	3238	34.5	2.53 ± 2.36	2.68 ± 1.45	5.21 ± 2.73
> 0 - ≤ 10.7	3073	32.7	2.88 ± 2.27	2.79 ± 1.46	5.67 ± 2.64
> 10.7	3073	32.7	2.74 ± 2.22	2.84 ± 1.49	5.58 ± 2.61

*h* hours, *ITE* Institute of Technical Education, *Kcal* Kilocalorie, *LTPA* Leisure Time Physical Activity, *MET* Metabolic Equivalent of Task, *NTC* National Technical Certificate, *O/N* O and N levels, *PSLE* Primary School Leaving Exam, 12 years old, *Q*, *SD* Standard Deviation

Across the entire sample, mean occupational sitting time was 2.71 (SD 2.29) hours a day, mean leisure sitting time was 2.77 (SD 1.47) hours a day and mean total sitting time was 5.48 (SD 2.67) hours a day. The average total working time among all participants was 8.68 (SD 2.41) hours a day. For each of the three ethnicities, the average total working time was relatively similar: 8.67 (SD 2.29) hours a day for Chinese, 8.61 (SD 2.53) hours a day for Malays, and 8.73 (SD 2.54) hours a day for Indians (data not shown).

**Fig. 1:**
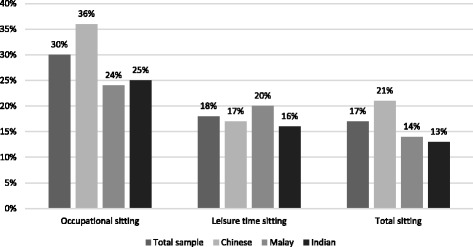
Proportion of the sample in the high sitting group, *p* < 0.01 for all comparisons.The cut-off for high total sitting time was ≥8 h/day. The cut-off for high occupational and leisure sitting time was ≥4 h/day

Fig. 1 presents the proportion of the sample who were dichotomized in the high sitting group based on the pre-defined cut-offs in hours of sitting per day. Significant differences across ethnic groups were found for total, occupational and leisure sitting (*p* < 0.01 for all). Chinese participants had the largest percentage of ‘high sitters’ for occupational sitting time and total sitting time, while Malay participants had the largest percentage of ‘high sitters’ during leisure time.

Tables 2, 3 and 4 present associations of socio-demographics and lifestyle factors with occupational, leisure and total sitting time for the total sample and stratified by ethnicity. All findings presented in this section are derived from multivariable models. They reflect statistically significant results as compared to the respective reference category of each variable.

**Table 2:** Multivariable associations of socio-demographic characteristics and lifestyle factors with OCCUPATIONAL sitting time (h/day)

Occupational sitting time	Total sample	Chinese	Malay	Indian
	n = 9384	*n* = 4722	*n* = 2246	*n* = 2385
	*B (95% CI)* *p-value*	*B (95% CI)* *p-value*	*B (95% CI)* *p-value*	*B (95% CI)* *p-value*
Age				
21-30^a^	0	0	0	0
31–40	**0.19 (0.04,0.34) 0.012**	0.14 (−0.07,0.36) 0.194	**0.33 (0.05,0.61) 0.023**	0.06 (−0.24,0.35) 0.709
41–50	**0.40 (0.25,0.55) <0.001**	**0.41 (0.19,0.64) <0.001**	**0.39 (0.11,0.67) 0.007**	**0.29 (0.00,0.58) 0.048**
51–60	**0.35 (0.19,0.52) <0.001**	**0.36 (0.12,0.61) 0.004**	0.18 (−0.15,0.51) 0.276	**0.40 (0.08,0.72) 0.014**
61+	**0.44 (0.20,0.68) <0.001**	**0.37 (0.04,0.71) 0.029**	0.43 (−0.07,0.92) 0.090	**0.66 (0.17,1.15) 0.009**
Gender				
Male^a^	0	0	0	0
Female	**0.28 (0.18,0.37) <0.001**	**0.33 (0.20,0.47) <0.001**	**0.22 (0.01,0.42) 0.039**	0.17 (−0.03,0.38) 0.100
Ethnicity				
Chinese^a^	0	–	–	–
Malay	**−0.34 (−0.45,-0.23) <0.001**	–	–	–
Indian	**−0.46 (−0.56,-0.36) <0.001**	–	–	–
Marital Status				
Married^a^	0	0	0	0
Never Married	−0.01 (−0.14,0.11) 0.813	−0.01 (−0.17,0.15) 0.867	−0.02 (−0.28,0.24) 0.895	−0.07 (−0.33,0.19) 0.609
Separated/Divorced/Widowed	**−0.31 (−0.50,-0.13) <0.001**	−0.25 (−0.55,0.05) 0.103	−0.30 (−0.66,0.06) 0.098	**−0.40 (−0.72,-0.09) 0.012**
Highest education				
No Formal/PSLE^a^	0	0	0	0
Secondary/O/N/ITE/NTC	**0.88 (0.76,0.99) <0.001**	**1.01 (0.83,1.19) <0.001**	**0.52 (0.30,0.73) <0.001**	**1.02 (0.80,1.24) <0.001**
A’level/Polytech/Diploma	**1.31 (1.17,1.46) <0.001**	**1.47 (1.26,1.68) <0.001**	**1.07 (0.78,1.37) <0.001**	**1.26 (0.98,1.54) <0.001**
University and above	**1.78 (1.63,1.94) <0.001**	**1.98 (1.76,2.20) <0.001**	**1.51 (1.05,1.97) <0.001**	**1.53 (1.23,1.84) <0.001**
Smoking				
No^a^	0	0	0	0
Former	−0.04 (−0.20,0.12) 0.619	−0.18 (−0.41,0.04) 0.113	0.30 (−0.01,0.60) 0.059	−0.08 (−0.42,0.26) 0.646
Yes	**−0.20 (−0.33,-0.08) <0.001**	**−0.21 (−0.40,-0.03) 0.024**	−0.21 (−0.42,0.01) 0.063	−0.20 (−0.44,0.03) 0.093
Total Caloric Intake (Kcal/day)				
1st quartile^a^	0	0	0	0
2nd quartile	0.11 (−0.01,0.23) 0.070	**0.19 (0.03,0.35) 0.020**	0.14 (−0.10,0.37) 0.257	−0.14 (−0.39,0.12) 0.299
3rd quartile	**0.27 (0.15,0.39) <0.001**	**0.29 (0.13,0.46) <0.001**	**0.42 (0.18,0.66) <0.001**	0.05 (−0.20,0.30) 0.717
4th quartile	**0.28 (0.16,0.40) <0.001**	**0.32 (0.15,0.49) <0.001**	**0.37 (0.14,0.61) 0.002**	0.05 (−0.20,0.29) 0.720
Moderate-to-vigorous LTPA (MET-h/week)				
0^a^	0	0	0	0
> 0 - ≤ 10.7	0.10 (−0.01,0.20) 0.070	**0.16 (0.02,0.31) 0.030**	−0.04 (−0.24,0.17) 0.718	0.05 (−0.16,0.26) 0.660
> 10.7	0.00 (−0.10,0.11) 0.970	0.07 (−0.09,0.22) 0.398	**−0.31 (−0.52,-0.10) 0.004**	0.12 (−0.09,0.32) 0.274

Bold numbers represent statistically significant findings

All models are corrected for total working time in hours per day

*h* hours, *ITE* Institute of Technical Education, *Kcal* Kilocalorie, *LTPA* Leisure Time Physical Activity, *MET* Metabolic Equivalent of Task, *NTC* National Technical Certificate, *O/N* O and N levels, *PSLE* Primary School Leaving Exam, 12 years old, *B* Beta, *CI* Confidence Interval

^a^reference category

**Table 3:** Multivariable associations of socio-demographic characteristics and lifestyle factors with LEISURE sitting time (h/day)

Leisure sitting time	Total sample	Chinese	Malay	Indian
	n = 9384	n = 4722	n = 2385	n = 2246
	*B (95% CI)* *p-value*	*B (95% CI)* *p-value*	*B (95% CI)* *p-value*	*B (95% CI)* *p-value*
Age				
21-30^a^	0	0	0	0
31–40	**−0.19 (−0.29,-0.09) <0.001**	**−0.20 (−0.35,-0.05) 0.010**	**−0.38 (−0.58,-0.18) <0.001**	0.03 (−0.18,0.24) 0.771
41–50	**−0.35 (−0.46,-0.25) <0.001**	**−0.33 (−0.49,-0.17) <0.001**	**−0.71 (−0.91,-0.51) <0.001**	−0.05 (−0.26,0.15) 0.616
51–60	**−0.33 (−0.44,-0.21) <0.001**	**−0.36 (−0.53,-0.19) <0.001**	**−0.50 (−0.74,-0.27) <0.001**	−0.06 (−0.29,0.16) 0.587
61+	**−0.51 (−0.68,-0.34) <0.001**	**−0.40 (−0.64,-0.17) <0.001**	**−0.89 (−1.24,-0.53) <0.001**	**−0.38 (−0.73,-0.04) 0.029**
Gender				
Male^a^	0	0	0	0
Female	**−0.11 (−0.17,-0.04) 0.003**	**−0.13 (−0.22,-0.04) 0.005**	−0.05 (−0.20,0.09) 0.474	−0.10 (−0.25,0.04) 0.163
Ethnicity				
Chinese^a^	0	–	–	–
Malay	−0.01 (−0.09,0.07) 0.815	–	–	–
Indian	**−0.18 (−0.26,-0.11) <0.001**	–	–	–
Marital Status				
Married^a^	0	0	0	0
Never Married	**0.20 (0.11,0.28) <0.001**	**0.21 (0.09,0.32) <0.001**	0.08 (−0.11,0.27) 0.400	**0.23 (0.04,0.41) 0.016**
Separated/Divorced/Widowed	**0.17 (0.04,0.30) 0.012**	0.05 (−0.16,0.26) 0.672	**0.28 (0.03,0.54) 0.031**	0.18 (−0.05,0.4) 0.118
Highest education				
No Formal/PSLE^a^	0	0	0	0
Secondary/O/N/ITE/NTC	−0.04 (−0.12,0.04) 0.313	0.01 (−0.11,0.14) 0.846	−0.11 (−0.26,0.05) 0.174	−0.04 (−0.19,0.12) 0.646
A’level/Polytech/Diploma	−0.03 (−0.13,0.08) 0.612	0.09 (−0.05,0.24) 0.207	0.00 (−0.21,0.21) 0.972	**−0.23 (−0.43,-0.03) 0.023**
University and above	−0.06 (−0.17,0.05) 0.266	0.07 (−0.08,0.22) 0.384	−0.02 (−0.35,0.31) 0.915	**−0.27 (−0.48,-0.06) 0.012**
Smoking				
No^a^	0	0	0	0
Former	0.05 (−0.06,0.16) 0.375	0.03 (−0.13,0.19) 0.716	0.20 (−0.02,0.42) 0.069	−0.04 (−0.28,0.20) 0.762
Yes	**0.20 (0.12,0.29) <0.001**	**0.15 (0.02,0.28) 0.028**	**0.28 (0.13,0.44) <0.001**	**0.19 (0.03,0.36) 0.023**
Total Caloric Intake (Kcal/day)				
1st quartile^a^	0	0	0	0
2nd quartile	−0.01 (−0.09,0.07) 0.849	−0.03 (−0.14,0.08) 0.609	0.02 (−0.15,0.19) 0.816	−0.02 (−0.20,0.16) 0.840
3rd quartile	0.05 (−0.03,0.13) 0.226	0.10 (−0.02,0.21) 0.096	−0.06 (−0.23,0.11) 0.522	0.05 (−0.13,0.23) 0.583
4th quartile	**0.10 (0.01,0.18) 0.025**	0.11 (−0.01,0.23) 0.067	0.02 (−0.15,0.18) 0.830	0.12 (−0.06,0.30) 0.180
Moderate-to-vigorous LTPA (MET-h/week)				
0^a^	0	0	0	0
> 0 - ≤ 10.7	**0.09 (0.01,0.16) 0.019**	0.03 (−0.08,0.13) 0.595	0.11 (−0.04,0.25) 0.154	**0.16 (0.01,0.31) 0.031**
> 10.7	0.07 (−0.01,0.14) 0.088	0.01 (−0.10,0.12) 0.873	0.04 (−0.11,0.19) 0.595	0.14 (0.00,0.29) 0.058

Bold numbers represent statistically significant findings

All models are corrected for total working time in hours per day

*h* hours, *ITE* Institute of Technical Education, *Kcal* Kilocalorie, *LTPA* Leisure Time Physical Activity, *MET* Metabolic Equivalent of Task, *NTC* National Technical Certificate, *O/N* O and N levels, *PSLE* Primary School Leaving Exam, 12 years old, *B* Beta, *CI* Confidence Interval

^a^reference category

**Table 4:** Multivariable associations of socio-demographic characteristics and lifestyle factors with TOTAL sitting time (h/day)

Total sitting time	Total sample	Chinese	Malay	Indian
	n = 9384	n = 4722	n = 2385	n = 2246
	*B (95% CI)* *p-value*	*B (95% CI)* *p-value*	*B (95% CI)* *p-value*	*B (95% CI)* *p-value*
Age				
21-30^a^	0	0	0	0
31–40	0.00 (−0.18,0.18) 0.991	−0.06 (−0.32,0.21) 0.671	−0.05 (−0.40,0.29) 0.768	0.09 (−0.28,0.45) 0.641
41–50	0.04 (−0.14,0.22) 0.656	0.08 (−0.19,0.36) 0.549	−0.32 (−0.67,0.02) 0.066	0.24 (−0.12,0.60) 0.191
51–60	0.02 (−0.18,0.23) 0.811	0.00 (−0.30,0.30) 0.992	−0.32 (−0.72,0.08) 0.117	0.34 (−0.06,0.73) 0.093
61+	−0.07 (−0.36,0.22) 0.626	−0.03 (−0.44,0.38) 0.879	−0.46 (−1.06,0.15) 0.139	0.27 (−0.33,0.88) 0.377
Gender				
Male^a^	0	0	0	0
Female	**0.17 (0.05,0.29) 0.005**	**0.20 (0.04,0.36) 0.015**	0.16 (−0.09,0.42) 0.204	0.07 (−0.19,0.32) 0.595
Ethnicity				
Chinese^a^	0	–	–	–
Malay	**−0.35 (−0.48,-0.21) <0.001**	–	–	–
Indian	**−0.64 (−0.77,-0.52) <0.001**	–	–	–
Marital Status				
Married^a^	0	0	0	0
Never Married	**0.18 (0.03,0.33) 0.017**	0.19 (0.00,0.39) 0.054	0.06 (−0.26,0.38) 0.702	0.16 (−0.17,0.48) 0.340
Separated/Divorced/Widowed	−0.14 (−0.37,0.08) 0.212	−0.20 (−0.57,0.16) 0.271	−0.02 (−0.46,0.42) 0.925	−0.23 (−0.62,0.17) 0.259
Highest education				
No Formal/PSLE^a^	0	0	0	0
Secondary/O/N/ITE/NTC	**0.83 (0.69,0.98) <0.001**	**1.02 (0.80,1.24) <0.001**	**0.41 (0.15,0.67) 0.002**	**0.98 (0.71,1.25) <0.001**
A’level/Polytech/Diploma	**1.29 (1.11,1.46) <0.001**	**1.57 (1.31,1.82) <0.001**	**1.07 (0.71,1.44) <0.001**	**1.03 (0.69,1.38) <0.001**
University and above	**1.72 (1.53,1.91) <0.001**	**2.05 (1.78,2.32) <0.001**	**1.50 (0.93,2.06) <0.001**	**1.26 (0.89,1.64) <0.001**
Smoking				
No^a^	0	0	0	0
Former	0.01 (−0.19,0.21) 0.917	−0.15 (−0.43,0.12) 0.274	**0.50 (0.12,0.88) 0.009**	−0.12 (−0.54,0.31) 0.588
Yes	0.00 (−0.15,0.15) 0.975	−0.07 (−0.29,0.16) 0.555	0.08 (−0.19,0.34) 0.579	−0.01 (−0.30,0.28) 0.948
Total Caloric Intake (Kcal/day)				
1st quartile^a^	0	0	0	0
2nd quartile	0.10 (−0.04,0.24) 0.171	0.16 (−0.03,0.35) 0.106	0.16 (−0.13,0.45) 0.289	−0.16 (−0.47,0.16) 0.341
3rd quartile	**0.32 (0.18,0.47) <0.001**	**0.39 (0.19,0.59) <0.001**	**0.36 (0.07,0.65) 0.015**	0.10 (−0.21,0.41) 0.545
4th quartile	**0.38 (0.23,0.53) <0.001**	**0.43 (0.23,0.64) <0.001**	**0.39 (0.11,0.68) 0.007**	0.17 (−0.14,0.47) 0.293
Moderate-to-vigorous LTPA (MET-h/week)				
0^a^	0	0	0	0
> 0 - ≤ 10.7	**0.18 (0.06,0.31) 0.005**	**0.19 (0.01,0.37) 0.036**	0.07 (−0.18,0.32) 0.592	0.21 (−0.05,0.47) 0.115
> 10.7	0.07 (−0.06,0.20) 0.312	0.08 (−0.11,0.26) 0.431	**−0.27 (−0.53,-0.01) 0.040**	0.26 (0.00,0.51) 0.050

Bold numbers represent statistically significant findings

All models are corrected for total working time in hours per day

*h* hours, *ITE* Institute of Technical Education, *Kcal* Kilocalorie, *LTPA* Leisure Time Physical Activity, *MET* Metabolic Equivalent of Task, *NTC* National Technical Certificate, *O/N* O and N levels, *PSLE* Primary School Leaving Exam, 12 years old, *B* Beta, *CI* Confidence Interval

^a^reference category

**Table 5:** Multivariable associations of socio-demographic characteristics and lifestyle factors with high sitting time

	High occupational sitting time^a^	High leisure sitting time^b^	High total sitting time^b^
	*OR (95% CI)* *p-value*	*OR (95% CI)* *p-value*	*OR (95% CI)* *p-value*
Age			
21-30^c^	1	1	1
31–40	1.03 (0.87,1.21) 0.749	**0.75 (0.63,0.90) 0.002**	0.93 (0.78,1.13) 0.475
41–50	**1.31 (1.11,1.54) 0.002**	**0.66 (0.55,0.79) <0.001**	1.01 (0.83,1.22) 0.920
51–60	1.16 (0.96,1.40) 0.116	**0.76 (0.62,0.94) 0.011**	1.02 (0.82,1.26) 0.882
61+	1.22 (0.91,1.63) 0.193	**0.63 (0.46,0.86) 0.003**	0.84 (0.59,1.20) 0.336
Gender			
Male^c^	1	1	1
Female	**1.77 (1.59,1.98) <0.001**	0.90 (0.79,1.03) 0.117	**1.18 (1.04,1.34) 0.012**
Ethnicity			
Chinese^c^	1	1	1
Malay	**0.74 (0.65,0.84) <0.001**	1.04 (0.91,1.20) 0.567	**0.70 (0.60,0.81) <0.001**
Indian	**0.67 (0.59,0.75) <0.001**	**0.85 (0.74,0.98) 0.022**	**0.55 (0.47,0.63) <0.001**
Marital Status			
Married^c^	1	1	1
Never Married	0.98 (0.85,1.12) 0.739	**1.25 (1.08,1.45) 0.004**	1.16 (0.99,1.35) 0.063
Separated/Divorced/Widowed	**0.75 (0.60,0.95) 0.018**	**1.26 (1.00,1.60) 0.048**	0.91 (0.69,1.20) 0.489
Highest education			
No Formal/PSLE^c^	1	1	1
Secondary/O/N/ITE/NTC	**2.44 (2.09,2.85) <0.001**	**0.83 (0.71,0.96) 0.015**	**1.50 (1.26,1.80) <0.001**
A’level/Polytech/Diploma	**3.46 (2.90,4.14) <0.001**	0.86 (0.72,1.04) 0.125	**1.81 (1.47,2.23) <0.001**
University and above	**5.74 (4.75,6.94) <0.001**	0.92 (0.75,1.13) 0.414	**2.55 (2.06,3.16) <0.001**
Smoking			
No^c^	1	1	1
Former	0.88 (0.73,1.06) 0.178	1.22 (0.99,1.49) 0.064	1.03 (0.84,1.28) 0.754
Yes	**0.80 (0.70,0.93) 0.003**	**1.57 (1.35,1.82) <0.001**	1.03 (0.87,1.21) 0.740
Total Caloric Intake (Kcal/day)			
1st quartile	1	1	1
2nd quartile	**1.17 (1.02,1.34) 0.024**	**1.25 (1.06,1.47) 0.008**	1.18 (1.00,1.40) 0.050
3rd quartile	**1.41 (1.23,1.62) <0.001**	**1.44 (1.22,1.69) <0.001**	**1.59 (1.35,1.87) <0.001**
4th quartile	**1.43 (1.24,1.64) <0.001**	**1.68 (1.43,1.97) <0.001**	**1.60 (1.36,1.88) <0.001**
Moderate-to-vigorous LTPA (MET-h/week)			
0^c^	1	1	1
> 0 - ≤ 10.7	1.04 (0.93,1.17) 0.481	1.07 (0.93,1.22) 0.362	1.03 (0.90,1.18) 0.683
> 10.7	0.95 (0.84,1.07) 0.370	1.08 (0.94,1.24) 0.295	0.98 (0.85,1.13) 0.758

Bold numbers represent statistically significant findings

All models are corrected for total working time in hours per day

*h* hours, *ITE* Institute of Technical Education, *Kcal* Kilocalorie, *LTPA* Leisure Time Physical Activity, *MET* Metabolic Equivalent of Task, *NTC* National Technical Certificate, *O/N* O and N levels, *PSLE* Primary School Leaving Exam, 12 years old, *OR* Odds Ratio, *CI* Conficende Interval

^a^Dichotomized as <4 h/day versus ≥4 h/day

^b^Dichotomized as <8 h/day versus ≥8 h/day

^c^reference category

### Correlates of occupational sitting time

In the total sample, those aged 31 years and above, females, those with at least a secondary level education and a daily Kcal intake in the 3rd and 4th quartile had higher occupational sitting time. Participants of Malay and Indian ethnicity, who were separated, divorced or widowed and smokers had lower occupational sitting time (Table 2).

Stratified analyses (Table 2) showed that Chinese and Indians aged 41 years and above had higher occupational sitting time, whereas in Malays, only those aged between 31 and 50 years had higher occupational sitting time. Chinese and Malay females had higher occupational sitting time. Having a secondary level education or higher was consistently associated with higher occupational sitting time across all ethnic groups. Higher daily Kcal intake was associated with higher occupational sitting time in Chinese (all quartiles) and Malays (3rd and 4th quartile). Chinese participants who engaged in up to 10.7 MET-h/week of LTPA had higher occupational sitting time.

Indians who were separated, divorced or widowed had lower occupational sitting time. Chinese smokers had lower occupational sitting time. Malays who engaged in more than 10.7 MET-h/week of LTPA had lower occupational sitting time.

### Correlates of leisure sitting time

In the total sample, those who were never married or separated, divorced or widowed, smokers, those with a daily Kcal intake in the 4th quartile, and who engaged in up to 10.7 MET-h/week of LTPA had higher leisure sitting time. Those aged 31 years and above, females and those of Indian ethnicity had lower leisure sitting time (Table 3).

Stratified analyses (Table 3) showed that Chinese and Indians who never married had higher leisure sitting time, whereas in Malays, being separated, divorced or widowed was associated with higher leisure sitting time. Smoking was consistently associated with higher leisure sitting time across all ethnic groups. Indians who engaged in up to 10.7 MET-h/week of LTPA had higher leisure sitting time.

Chinese and Malays aged 31 years and above had lower leisure sitting time, whereas in Indians, only being aged 61+ years was associated with lower leisure sitting time. Chinese females had lower leisure sitting time. Indians with an A’level education or higher had lower leisure sitting time.

### Correlates of total sitting time

In the total sample, females, those who were never married, with a secondary level education or higher, a daily Kcal intake in the 3rd and 4th quartile, and who engaged in up to 10.7 MET-h/week of LTPA had higher total sitting time. Participants who were of Malay and Indian descent had lower total sitting time (Table 4).

Stratified analyses (Table 4) showed that Chinese females had higher total sitting time. Having a secondary level education or higher was consistently associated with higher total sitting time across all ethnic groups. Indians who were former smokers had higher total sitting time. Chinese and Malays with a daily Kcal intake in the 3rd and 4th quartile had higher total sitting time. Chinese who engaged in up to 10.7 MET-h/week of LTPA had higher total sitting time.

Malays who engaged in more than 10.7 MET-h/week of LTPA had lower total sitting time.

Table 5 presents associations of socio-demographics and lifestyle factors with high sitting time for the total sample. All subsequent findings are derived from multivariable models. They reflect statistically significant results as compared to the respective reference category of each variable.

### Correlates of high sitting time

Higher odds for high occupational sitting time were found for those aged between 41 and 50 years, females, those with a at least a secondary level education, and a higher daily Kcal intake (all quartiles). Lower odds for high occupational sitting time were found for those of Malay and Indian descent, who were separated, divorced or widowed and smokers.

Higher odds for high leisure sitting time were found for those who were never married, separated, widowed or divorced, smokers and those with a higher daily Kcal intake (all quartiles). Lower odds for high leisure sitting time were found for being aged 31 years and above, those of Indian descent and with a secondary level education.

Higher odds for high total sitting time were found for females, those with at a secondary level education or higher, and those with a daily Kcal intake in the 3rd and 4th quartile. Lower odds for high total sitting time were found for Malays and Indians.

### Robustness of the associations

When the multivariable models were repeated including only participants who worked full-time, most of the associations between correlates and sitting time remained unchanged, with a few exceptions (data not shown). Most importantly, patterns for age and occupational sitting became less clear as certain age groups in the Chinese (61+ years), Malay (31–40 years) and Indian (41–50 years) samples were no longer significantly associated with higher occupational sitting time. Also, the association between smoking and higher leisure time sitting among Indians disappeared, which is contrasting to results for the combined and Chinese/Malay samples. Regarding LTPA engagement, the association with higher occupational and total sitting time among Chinese and the association with lower total sitting time among Malays disappeared, whereas among the combined sample an additional significant association with leisure time sitting was found, and among Indians an additional significant association was found for both higher leisure time and higher total sitting time. These changes eventually resulted in a clearer pattern across sitting domains and ethnicities: LTPA engagement was associated with higher leisure time sitting time and total sitting time for the combined sample as well as among Indians. In the dichotomous analyses, associations of age with occupational sitting seemed to become more consistent, as those aged 51–60 years and 61+ years had significantly higher odds to have high occupational sitting time. Yet, for those aged 51–60 years higher odds to have high leisure time sitting disappeared, which is in contrast to the findings for other age categories. The above findings should be interpreted in light of the smaller sample size that was used for testing the robustness of our original results, which may possibly have caused some of the changes in significance levels.

## Discussion

Findings from this study improve the understanding of socio-demographic and lifestyle factors that are associated with sitting time in an understudied population, namely a multi-ethnic sample from Singapore, including Chinese, Malays and Indians. The MEC data allowed us to map out correlates of sitting time for occupational, leisure and total sitting and to look at differences between ethnicities.

Our participants reported to sit about 5 ½ hours a day on average, with the highest proportion of ‘high sitters’ (i.e., > 8 h a day) being of Chinese ethnicity. These findings are in line with another recent cross-sectional study including Singapore adults, which showed that they had a median sitting time of 6 h on a typical day, and also that Chinese had the highest prevalence of high sitters [10]. In addition, Sloan and colleagues [28], who analyzed data from the latest Singapore Ministry of Health’s National Health Survey, reported a slightly lower sitting time of 5 h a day among Singapore adults aged 18 to 79 years. In this study, Chinese were found to have higher sitting time than Malays and Indians too.

Compared to neighboring country Malaysia, our Singapore sample seems to spend less time sitting. A study by Chu and Moy [29] showed that Malay adults sat between 7.4 and 7.7 h a day on average. However, unlike in the current study, the assessment of total sitting time included sitting for transport. Another study among Korean adults also showed a higher overall sitting time of about 7 h a day [30], whereas in a Japanese sample self-reported average sitting time was even higher: 8.4 h a day [5]. Similar to our assessment of sitting time, neither of these two studies took sitting for transport into consideration.

Compared to several other Asian countries the proportion of high sitters in our sample was relatively low, i.e., 17%. In Japan, Hong Kong and Taiwan already 25% of the investigated adults was estimated to sit 9 or more hours a day (values based on self-report) [31]. Yet, in China and India only 7.6% and 6.3% respectively reported to sit that much, with the largest proportion of people sitting between 3 and 4 h a day (China; 29.8%) and less than 3 h per day (India; 39.2%) [31]. From a more international perspective, our data suggests that Singapore would rank middle-to-high in terms of total sitting time when compared to data from over 32 European countries [32]. However, it must be noted that prevalence estimates can vary greatly depending on the utilized self-report assessment method [33].

Overall, occupational sitting and leisure time sitting seemed to contribute equally to total sitting time. This finding is in contrast with another recent study among Singapore adults which reported a median of 7 h spent sitting at work, and a median of 5 ½ hours a day spent sitting during leisure time [34]. Similarly, a study in Australian adults using self-reported sitting time estimated that occupational sitting accounted for almost 60% of total sitting time [11]. An important difference between the current study and the ones conducted by Waters et al. [34] and Bennie et al. [11] is that we did not only include office-based workers. Our sample is more diverse as it comprises participants with different job types. Also, both part-time and full-time workers were included in the analyses, which may have led to lower daily estimates of occupational sitting than for samples including full-time workers only. Nevertheless, those with higher education tended to accumulate the highest occupational sitting time, probably reflecting having an office-based employment.

### Correlates of sitting

Our results on correlates of sitting across the entire sample are generally in line with those reported in the systematic review of O’Donoghue and colleagues [19] in which evidence was presented for associations of older age, being female and smoking with higher sitting time. O’Donoghue et al. [19] also considered diet to be associated with higher sitting time, though the studies that were included in the review assessed consumption of high calorie snacks and not daily caloric intake. According to the authors, associations of marital status with sitting time were mixed, while our analyses showed that those who were never married, compared to married participants, had higher sitting time. They also mentioned that socio-economic status was possibly the most consistent indicator of sitting. Likewise, betas of educational level on sitting time were relatively large among our sample compared to those of other explanatory variables. Overall, it may be concluded that the socio-demographic and lifestyle factors that influence sitting time in an Asian population, are fairly similar to those in more Western-oriented populations. One exception is the engagement in LTPA, which we found to be associated with higher sitting time instead of lower sitting time but associations were inconsistent with small betas. For overall physical activity, O’Donoghue [19] and colleagues reported that the majority of included studies found an inverse association with sitting. It may be that those who sit more try to compensate for this by engaging in greater LTPA. Alternatively, higher education could be partly responsible for the association between LTPA and higher sitting time, which would be consistent with findings across the European region showing that higher socio-economic status (mainly derived from education level) is correlated with both higher sitting time and higher LTPA engagement [35]. Generally, we would like to highlight that the observed differences in prevalence and correlates of sitting time between our study and the (cohort) studies described above may be due to other factors than those already mentioned. Possible influential factors include the type of sample analysed (representative sample versus longitudinal/cross-sectional cohorts), differences in population characteristics (multi-ethnic versus primarily one ethnicity), the way occupational sitting time was calculated and expressed, and also the fact that Singapore being a city state with a very consistent environment may have less diversity in the environment individuals are exposed to compared to larger countries.

### Domain-specific sitting

One of the most important features of our study is the ability to distinguish between the associations of socio-demographic and lifestyle factors with occupational and leisure time sitting, showing associations in opposite directions for both sitting domains. Our analyses showed that these associations were highly depending on the domain of sitting assessed. For example, those of older age spent more time in occupational sitting, but less time in leisure time sitting. Women engaged in higher occupational and total sitting time, but in lower leisure sitting time, compared to men. For those who were separated, widowed or divorced, or smokers, the association pointed in the opposite direction: less occupational sitting time, but more leisure sitting time. Educational level and engaging in LTPA were associated with higher sitting, except for leisure time sitting and occupational sitting, respectively. These findings highlight the need to focus on separate domains of sitting when trying to identify determinants of this behavior, as different sitting time domains might be associated with different factors. Recently validated self-report instruments offer the opportunity to assess sitting in different contexts such as at work, during travel, while watching TV (for leisure), while using the computer (for leisure), with the possibility to further focus on week- versus weekend days or working- versus non-working days [36–38]. Busschaert et al. [39] evaluated an even more detailed context-specific sedentary behavior questionnaire, which also included sitting to e.g., care for (grand) children, making phone calls and/or having meals. Researchers are encouraged to use more comprehensive tools for the identification of important factors influencing sitting time. Yet, it should be acknowledged that such tools may come at the expense of ‘higher’ assessed sitting levels, making comparisons to previous studies more difficult.

In addition, our findings indicate that the content of intervention programs should be tailored to domain-specific sitting rather than to sitting in general. Around the globe, interest in such approaches is increasing. For example, a review summarized 20 controlled trials which tested the effect of interventions to reduce sitting time at work, e.g., the use of sit-stand desks or treadmill work stations, providing computer prompts, and/or counselling [40]. Although the database search was not limited to an earliest publication date, all studies were conducted relatively recently, between 2009 and 2015. Yet, the authors concluded that there is a lack of evidence for the effectiveness of any type of intervention in the workplace, and they urged researchers to conduct cluster-randomised trials with a sufficient sample size and long-term follow-up to tackle this issue. Another systematic review and meta-analyses presented a more positive picture i.e., when pooling the intervention effects of selected studies, a significant reduction in workplace sitting for intervention groups was found. Interventions including multiple components such as education plus environmental changes reported the greatest decrease in sitting time [41]. In this context, one critical – but missing from the current study – component influencing occupational sitting time is job type. Previous research has repeatedly shown an association of white collar/professional occupations with higher occupational sitting time [42–44]. Future studies on sedentary behavior should account for this.

### Ethnic differences

All multivariable models showed that Malay and Indian participants had lower occupational, leisure and/or total sitting time than Chinese participants; i.e., Chinese participants were most sedentary.

Our analyses revealed additional differences between ethnic groups in terms of influential socio-demographic and lifestyle factors of sitting that warrant attention. Factors associated with occupational, leisure and total sitting time in Indians seemed different to those found in Chinese and Malays. Chinese and Malay females spend more time in occupational sitting than their male counter parts, while this was not the case for Indian females. Daily caloric intake was associated with higher occupational sitting time among Chinese and Malays, but not among Indians. Regarding leisure time sitting, only the highest age group (61+ years) had lower sitting time in Indians, whereas for Chinese and Malays, all those aged over 30 had lower sitting time compared to those aged under 30. Indians were also the only ethnic group showing an association between higher education levels and lower leisure sitting time. Culturally appropriate health promotion programs seem to be more effective than usual care or other control conditions [45]. The so-called ‘cultural targeting’ of health promotion programs can be achieved in many ways, for example by providing project materials in participants’ native language or showing participants the impact of a certain health problem on their ethnic group [46]. In light of our findings, we suggest for the multi-ethnic Singapore context to consider Indians as a separate target group when developing interventions aiming to reduce sitting time. Both in terms of language, as well as in terms of the possible influential factors and sitting domain(s) it targets. Generally, we recommend cultural targeting of interventions when suitable, and also the oversampling of minority groups when recruiting participants for cohort studies, like in the MEC. It would likely increase the involvement and participation of members of the minority cultural groups, which are often the ones who are in need of intervening and/or for whom less information on determinants of health behavior is available.

### Continuous versus dichotomous outcomes of sitting

When sitting time was analyzed as a dichotomous outcome, results were fairly comparable to the results from the multivariable models with a continuous outcome. Yet, significant associations of most age groups with occupational sitting time disappeared. This was also the case for significant associations of gender and LTPA with leisure sitting time, and marital status and LTPA with total sitting time. Additional significant associations were found for educational level and leisure sitting time (those with a secondary education had higher odds to be in the ‘high’ sitting group) and diet and occupational and leisure sitting time (those with higher daily caloric intake had higher odds to be in the ‘high’ sitting group). It is generally accepted that sitting in itself is not a problem, but high levels of sitting are. Hence, the associations from dichotomous analyses may be more informative for intervention development. Yet, it has to be noted that there is no consensus on the cut-off for sitting too much, nevertheless we used a cut-off from a recent meta-analysis, which provides one of the first indications of how much sitting might be considered unhealthy [2]. Researchers should take into account that such cut-offs are highly influenced by assessment methods, as e.g., accelerometry and domain-specific sitting questionnaires will give substantially higher sitting estimates than single item sitting questions such as those used in our study.

#### Strengths and limitations

Among this study’s major strengths are the relatively large sample size and the ability to assess correlates of sitting among multiple Asian ethnic groups. Further, we examined correlates of domain-specific sitting, i.e. occupational, leisure and total sitting time, providing a more refined summary of the differences in important influential factors for different domains of sitting. Finally, all our statistical models were corrected for participants’ total working time, as those who work more theoretically have more time to sit during work and less time to sit during leisure time. We also acknowledge the following limitations. Firstly, the MEC only provided information on leisure sitting and occupational sitting, from which we calculated total sitting time. Many Singaporeans use Singapore’s efficient public transport system on a daily basis, but sitting for transport was not assessed in the MEC. This may have resulted in an underestimation of total sitting time. Yet, a recent study among Singapore office-based workers estimated that only less than 10% of total sitting time came from sitting during transport [34]. Secondly, information on sitting time was self-reported, which is susceptible to social desirability and recall bias. Thirdly, other characteristics of sitting time such as number of sit-stand transitions and prolonged bouts of sitting were not captured as part of this study. They may, however, be differently associated with socio-demographic and lifestyle factors than accumulated sitting time. Fourthly, we were not able to account for job type, which is likely to be a correlate of especially occupational sitting time and its addition to the multivariate model might have influenced the betas of related constructs, such as level of education. Lastly, our results are based on a sample from Singapore and may therefore not be generalizable to other populations.

## Conclusion

In this multi-ethnic Singapore sample of working adults, occupational and leisure sitting time contributed equally to total sitting. Correlates of sitting time highly depended on the domain of sitting (occupational, leisure or total) and important differences between different ethnic groups were identified. Our findings highlight the need to focus on separate domains of sitting when trying to identify determinants of this behavior, and that the content of intervention programs should probably be tailored to domain-specific sitting rather than to sitting in general. Moreover, we recommend researchers to culturally target interventions when suitable.

## Abbreviations

BMI: Body Mass Index
FFQ: Food Frequency Questionnaire
ITE: Institute of Technical Education
Kcal: Kilocalorie
LTPA: Leisure Time Physical Activity
MEC: Singapore Multi Ethnic Cohort
MET: Metabolic Equivalent of Task
NTC: National Technical Certificate
O/N: O and N levels
PSLE: Primary School Leaving Exam, taken at 12 years old
SCCS2: Singapore Cardiovascular Cohort Study
SD: Standard Deviation
SP2: Singapore Prospective Study Program
SP2PAQ: SP2 Physical Activity Questionnaire
